# A hybrid vision transformer and ResNet18 based model for biotic rice leaf disease detection

**DOI:** 10.3389/fpls.2025.1711700

**Published:** 2025-11-14

**Authors:** Sankar Sennan, Ramasubbareddy Somula, Yongyun Cho, Selvaganapathi Sennan

**Affiliations:** 1 Department of Information and Communication Engineering, Sunchon National University, Suncheon, Republic of Korea; 2 Symbiosis Institute of Technology, Symbiosis International (Deemed University), Pune, India; 3 Technical Architect, Hexaware Technologies, Tempe, AZ, United States

**Keywords:** rice leaf disease, vision transformer, Resnet18, agriculture, image classification, deep learning, biotic rice, neural networks

## Abstract

**Introduction:**

Agriculture is crucial to human survival. The growing of biotic rice plants is very helpful for feeding a lot of people around the world, especially in places where rice is a main food. The detection of rice leaf disease is critical to increasing crop productivity.

**Methods:**

To improve the accuracy of rice leaf disease prediction, this paper proposes a hybrid Vision Transformer (ViT) with pre-trained ResNet18 models (ViT-ResNet18). In general, the input images apply to the pre-trained ViT and ResNet18 models independently. The output features of these two models are combined and fed into the final Fully Connected (FC) layer, followed by a Softmax layer for final classification.

**Results:**

The output of rice leaf diseases from the FC layer of the proposed hybrid ViT with ResNet18 model achieved 94.4% accuracy, a precision of 0.948, a recall of 0.944, an F1-Score of 0.942, and an Area Under Curve (AUC) of 0.985.

**Discussion:**

The proposed hybrid model ViT-ResNet18 shows a 5%, 1%, and 1% improvement in accuracy compared to VGG16 with Neural Network, Inception V3 with Neural Network, and SqueezeNet with Neural Network classifier, respectively.

## 1 Introduction

Agriculture is critical to both the economy and a country’s progress. In developing countries, agriculture is an important source of employment and money. The global population is projected to exceed 10 billion by 2050. Therefore, the need for agricultural products is always rising (Jafar et al., 2024). Rice is an imported food, and most countries consume it as rice. Biotic rice is a breed that is resistant to biological threats such as insects and fungi. Furthermore, rice crops are mostly produced throughout Asia and around the world. Due to population growth, farmers need to increase production by roughly 1.2% each year (Li et al., 2024). There are various reasons why the farmer was unable to meet the desired level of rice production. One of the causes is that rice leaves are susceptible to a variety of illnesses. Rice leaf diseases encompass leaf blast, bacterial blight, and brown spot, among others (Trinh et al., 2024; Simhadri et al., 2025).

Finding rice leaf disease is a challenging task. The reason is that disease detection is done manually, which takes longer, or the farmer notices it too late, does not identify the disease, and sometimes predicts the disease incorrectly. Earlier, a greater number of specialists and plant monitoring were required in manual inspection. However, the farmers were unable to obtain expert assistance due to a lack of facilities and an inability to absorb the cost. To overcome the challenges mentioned above and achieve automatic disease detection, a technology-based solution is needed to detect and recognize leaf illnesses promptly and give care based on the severity of the diseases (Kaur and Trivedi, 2024).

Earlier, Image Processing (IP) and Machine Learning (ML) were utilized to detect rice leaf diseases. IP is used to improve images and extract features, such as numerical values (Rahman et al., 2024). Rice leaf disease is predicted by using machine learning (ML) methods. Though ML algorithms have been used in disease prediction, their accuracy is not significant.

Deep Learning (DL) primarily relies on Artificial Neural Networks (ANN), which are employed to learn from data and facilitate decision-making, or disease classification based on images. The architecture has three separate layers: input, hidden, and output (Haridasan et al., 2023; Rajpoot et al., 2023). In the past few years, many studies have used DL algorithms to identify illnesses that affect rice leaves. The researchers focused on DL methods that are used a lot, like Convolutional Neural Networks (CNN) and pre-trained models based on Transfer Learning, such as ResNet, Inception Net, Vision Transformer (ViT), and others (Ahad et al., 2023; Simhadri and Kondaveeti, 2023a; Thai et al., 2023).

Single DL approaches did not achieve higher accuracy. To improve classification accuracy, the researchers used a hybrid DL model. In general, hybrid DL models enhance accuracy while successfully dealing with local and global features (Chug et al., 2023). Recent studies show that hybrid DL models have been utilized for the identification of rice plant diseases; however, there is still an opportunity to improve prediction accuracy. As a result, this paper proposes a hybrid Vision Transformer (ViT) that incorporates pre-trained ResNet18 models. In general, the input images apply to the pre-trained ViT and ResNet18 models independently. The outputs from these two models are concatenated and subsequently input into the final FC layer. The output of rice leaf disease from the FC and Softmax layers.

The highlights of the contribution are follows:

- In the related works, several deep learning-based methods for detecting rice leaf disease were presented, and their shortcomings were identified.
- To improve the detection of biotic leaf disease, a hybrid novel model combining ViT and ResNet18 is proposed.
- We have adjusted the number of epochs to determine the best training configuration in order to improve the accuracy of leaf disease detection.
- To validate the effectiveness of the proposed hybrid model ViT- ResNet18 and its variant, an ablation study was conducted.
- The goal of this research work is to improve crop disease detection accuracy and promote food security through the use of deep learning models in agricultural applications.

The structure of the paper is outlined as follows: Section 2 provides an overview of the related work. Section 3 discusses deep learning architecture. Section 4 presents a hybrid Vision Transformer (ViT) with ResNet18 model. In Section 5, a comparative analysis of the performance of the proposed model against existing models is presented. Section 6 provides the analysis and discussion. The conclusion and future research are presented in the paper’s conclusion.

## 2 Related works

This section outlines numerous research works focusing on rice leaf disease prediction.

Kaur et al (Kaur et al., 2024). proposed a deep learning model-based rice leaf detecting method. This research employs pre-trained models to perform feature extraction, alongside DL models for the classification process. The evaluation of the feature extraction method was conducted utilizing pre-trained models, including VGG16, Inception V3, and Squeeze Net. The classification approach utilized ML and DL models, including SVM, Naïve Bayes, K-Nearest Neighbor (KNN), and neural networks. Following multiple experiments, this work came up with the Squeeze Net for feature extraction and neural networks for classification. It attained an accuracy of 93.3%. However, it is necessary to improve the accuracy of the disease-affected images.

Aggarwal et al (Aggarwal et al., 2023). proposed ML-based rice leaf prediction. Images are preprocessed according to image dataset model parameters. This work involves two processes: feature extraction and categorization. For feature extraction, 32 pre-trained deep learning models were employed, including VGG, ResNet, Inception V3, Xception, EfficentNet, and its variation. This work uses a variety of machine learning techniques for categorization. The primary emphasis was on rice leaf diseases, specifically leaf blast, bacterial blight and brown spot. The proposed model outperformed others. However, it has only considered three disease types and needs to improve its accuracy.

Simhadri and Kondaveeti (2023b) proposed a rice leaf detecting method utilizing transfer learning. This work used transfer learning on 15 pre-trained CNN models to identify rice leaf disease. It receives the input image and applies the resize operation. The dataset was split into training and testing. Data augmentation was performed for the class with fewer images. After conducting several experiments, Inception v3 outperformed other deep learning models. In total, ten classes were used in this research. However, the model exhibits increased misclassification for specific categories.

Lamba et al (Lamba et al., 2023b). proposed a fine-tuned deep learning model for paddy leaf disease classification. This work emphasizes paddy leaf disease severity. The dataset images were gathered from GitHub, UCI, Kaggle, and Mendeley. The dataset contains 4068 images. Initially, images are pre-processed using an image generator. A Generative Adversarial Network (GAN) was used to enhance the images. Leaf image severity is calculated using segmentation algorithms. CNN and SVM are feature extraction and classification methods. The diseases are classified as mild, moderate, severe, and profound. This model predicted a high severity rate compared to previous models. However, this model only tested bacterial blight, blast, and leaf smut.

Bijoy et alet al., 2024). proposed utilizing deep CNN (dCNN) to detect rice leaf diseases. This work collected an enhanced dataset of 5593 images from five disease types. Sheath blight, brown spot, bacterial leaf blight, and leaf smut are the four disease classifications. The input image has been scaled and the feature extracted before being applied to the pooling layers. The proposed dCNN’s performance is compared to standard benchmark models such as AlexNet, MobileNet, and ResNet50. However, it is important to increase the number of leaf disease categories.

Lamba et al (Lamba et al., 2023a). proposed a hybrid GCL model for paddy leaf diseases. This work utilizes datasets from Mendeley, UCI, Kaggle, and GitHub. It employed 3535 images across three classes: bacterial blight, rice blast, and leaf smuf. The GAN model is used for data augmentation. This hybrid work combined CNN and the Long-term Short Memory (LSTM) model. It increased accuracy by 97% when compared to previous approaches. However, it has only used three disease categories.

Abasi et al (Abasi et al., 2023). introduced an improved method for detecting rice leaf diseases utilizing a customized CNN model. This was developed specifically for images related to rice leaf disease. The input image dimensions are 224 × 224 x 3. The dataset is divided into three segments: 80% allocated for training, 10% designated for validation, and 10% reserved for testing. Features of rice leaves may be extracted from images using the CNN model. The customized CNN model includes convolution, max pooling, flattening, and dropout layers. This model scored a 91% accuracy against the Inception v3 and EfficientNet B2 models. This customized model delivered higher accuracy, lower loss, and reduced model overfitting. However, the rice leaf disease dataset’s accuracy need improvement.

Narmadha et al (Narmadha et al., 2022). proposed a method for detecting rice leaves utilizing deep learning techniques. The DenseNet169-MLP model combines a dense CNN (DenseNet) with a multi-layer perceptron (MLP). It seeks to accurately classify the leaf diseases. The dataset employed three classes: brown spot, bacterial leaf blight, and leaf smut. This process begins with channel separation, followed by greyscale conversion and noise removal with a median filter. The segmentation process is carried out utilizing fuzzy c methods. The DenseNet169 model, which has been pre-trained, is utilized for the purpose of feature extraction, whereas a Multi-Layer Perceptron (MLP) is applied for classification tasks. The proposed model’s performance is compared to other models. It outperforms other similar methods in terms of accuracy. However, it only analyzed three classifications in the input dataset and needs to improve accuracy. It also did not address the severity of diseases.

Upadhyay and Kumar (2022) proposed a DL algorithm for predicting rice plant leaf diseases. It focusses on the following diseases: leaf smut, leaf blight, and brown spot. Most disease predictions depend on leaf size, color, and shape. Otsu’s global thresholding removed image background noise. The proposed CNN was trained on healthy, leaf smut, leaf blight, and brown spot datasets. Each class contains 4000 photos. The proposed CNN outperformed other models. However, it must focus on improving the accuracy of leaf disease images.

Ramadan et al (Ramadan et al., 2025). built a model for rice leaf disease detection using CNNs and GANs. They aimed to solve the problem of small and imbalanced datasets by creating synthetic images. The main advantage of using GANs for leaf disease detection is their ability to generate synthetic images. This increases the dataset size, adds variety, and lowers the chance of overfitting. Three GAN types were tested: Simple GAN, CycleGAN, and DCGAN. CycleGAN gave the best results.

Swati et al (Lipsa et al., 2025). built a CNN model for rice leaf disease detection with a focus on interpretability. To address the black box issue in CNNs, they used three explainable AI tools: Layer-wise Relevance Propagation (LRP), SHAP, and LIME. LRP traced how each layer contributed, SHAP explained feature importance, and LIME showed the image regions that guided predictions. The proposed method reached 96.5% of accuracy.

Most existing models focus on only a few rice leaf disease types. Their accuracy is often limited. Many models misclassify diseases or overfit when trained on small datasets. Some use only CNNs or transfer learning and do not explore hybrid models for stronger feature extraction. This study proposes a hybrid model that joins a Vision Transformer (ViT) with ResNet18. ViT captures global image patterns, while ResNet18 extracts local details. Combining these features gives a richer representation. Our experiments show the hybrid model reaches 94.4% accuracy. It performs better than SqueezeNet with Neural Network, VGG16 with Neural Network, and InceptionV3 with Neural Network. The results show fewer misclassifications and higher accuracy in rice leaf disease detection. **Table 1**. summarizes the related works which covers the research articles contributions and limitations.

**Table 1:** Summary of related works and their contributions and limitations.

Authors	Proposed Model	Advantages	Limitation
Kaur et al (Kaur et al., 2024)	Feature Extraction: VGG16, Inception V3 and Squeeze Net Classification: SVM, KNN, NN	Gained 93.3% accuracy using transfer learning with standard classifiers.	Accuracy below 95% and weak results on complex disease images.
Aggarwal et al (Aggarwal et al., 2023)	Feature extraction-32 pre-trained deep learning models Classification- ML algorithms	Reached 91% accuracy using many pre-trained models on three diseases.	Only considered three classes, poor generalization to other diseases.
Simhadri and Kondaveeti (2023b)	15 pretrained CNN models	Better results across ten classes using transfer learning and augmentation.	High misclassification in unbalanced data, less reliable overall.
Lamba et al (Lamba et al., 2023b)	fine-tuned deep learning model	Classified disease severity into four levels, useful for field diagnosis.	Only focused on three diseases, lacks wider disease coverage.
Bijoy et al (Bitoy et al., 2024)	deep CNN	Tested on 5593 images, suitable for IoT-based low-resource systems.	Accuracy drops on manual datasets, limited scalability.
Lamba et al (Lamba et al., 2023a)	hybrid GCL model	Reached 97% accuracy by combining CNN and LSTM with GAN augmentation.	Only covered three classes, not scalable for large datasets.
Abasi et al (Abasi et al., 2023)	customized CNN model	Reduced overfitting and achieved 91% accuracy against strong baselines.	Still lower than hybrid models, needs better generalization.
Narmadha et al (Narmadha et al., 2022)	DenseNet with MLP	Boosted feature extraction and classification with DenseNet169+MLP.	Limited to three classes and ignored disease severity.
Upadhyay and Kumar (2022)	CNN model	Handled four disease types with better accuracy.	It only considered 3 classes, focused on the other dataset, and needs to increase the accuracy.
Ramadan et al (Ramadan et al., 2025)	CNN +GAN	Achieves high accuracy	GAN training is slow and resource heavy.
Swati et al (Lipsa et al., 2025)	Interpretable and Explainable CNN	Provides transparency with explainable AI tools and Shows image regions influencing predictions	Accuracy is lower than some GAN or hybrid models.

## 3 Deep learning architectures

This section covers the fundamental concepts and functionalities of the Vision Transformer (Han et al., 2022) and ResNet18 models (Ayyachamy et al., 2019). Section 4 discusses the proposed hybrid model.

### 3.1 Vision transformer

This model is designed for deep learning applications focused on image-related tasks, including image classification, segmentation, and object detection. The input image size (224 × 224 x 3) is typically fed into the Vision Transformer (ViT) model and is represented as Height x Width x Number of channels (Red, Green, and Blue).

#### 3.1.1 Input image

The image has been divided into patches of constant size, pt x pt (16 × 16) pixels. The number of patches (N) is computed using Equation 1.

(1) $$N=\frac{Height\;\times \;Width}{pt^{2}}$$

where pt is the patch size.

#### 3.1.2 Patch embedding

Each patch is converted to a one-dimensional (1-D) vector, which is represented in Equation 2.

(2) $$y_{i}=Flatten\left(pt_{n}\right)$$

Where $y_{i}$ denotes Flattened vector of i^th^ patch (pt).

The flattened patches are linearly converted using a learnable weight matrix, which is provided in Equation 3.

(3) $$z_{i}=w_{embed}\times y_{i}$$

Where $z_{i}$ denotes embedded vector of i^th^ patch (pt).

#### 3.1.3 Positional embedding

Transformers cannot capture position information directly. To preserve spatial order, positional encoding is added. The final embedding of the *i*^th^ patch ( $embed_{i}$) is calculated by combining the positional encoding of the *i*^th^ ( $pos_{i}$) with $z_{i}$ embedded vector of *i*^th^ patch, as shown in Equation 4.

(4) $$embed_{i}=z_{i}+pos_{i}$$

where $pos_{i}$ is the positional encoding of the i^th^ patch.

#### 3.1.4 Transformer encoder

The encoder takes the sequence of embeddings ( $embed_{1}$, $embed_{2}$,…… $embed_{n}$).

It consists of Multi-Head Self-Attention (MHSA), a Feed-Forward Network (FFN), and Layer Normalization (LN).

##### 3.1.4.1 Multi-head self-attention

It extracts or maintains global dependencies between patches. The self-attention computes the association between all patches (pt). For each head, Query (Q), Key (K), and Value (V) must be calculated, as shown in Equations 5–7.

(5) $$\;Q=embed\;\times \;w_{Q}\;$$

(6) $$K=embed\;\times \;w_{K}\;$$

(7) $$\;\;\;\;\;\;V=embed\;\times \;w_{V}\;$$

where embed indicates the path embedding, $w_{Q}$, $w_{K}$ and $w_{V}\;$ are learnable weight matrices.

In addition, it computes the attention score, which is represented in Equation 8.

(8) $$attention\;\left(Q,K,V\right)=Softmax\left(\frac{Q\;\times K^{T}}{\sqrt{dim_{K}}}\right)\times V$$

where $dim_{K}$ denotes the dimensionality of K.

Finally, ViT combines multiple heads and is defined in Equation 9.

(9) $$MHSA\left(embed\right)=concat\left(\;Head_{1},\;Head_{2}\ldots .Head_{n}\right)\times W_{output}$$

where $W_{output}$ denotes the output weight matrix.

##### 3.1.4.2 Feedforward neural networks

Each patch embedding is processed using a feed-forward neural network, and its calculation is provided in Equation 10.

(10) $$FFNs=ReLu\;\left(embed\;\times \;w_{1}+b_{1}\right)\times w_{2}+b_{2}$$

where $w_{1}$ and $w_{2}$ are weight matrix of first and second feed-forward networks, $b_{1}$ and $b_{2}$ are the bias vectors of the first and second feed-forward networks.

##### 3.1.4.3 Layer normalization and residual connections

It adds the residual connections to the training and is calculated using Equations 11 and 12.

(11) $$embed^{'}=LayerNorm(embed+MHSA\left(embed\right)$$

(12) $$embed^{''}=LayerNorm(embed+FFN\left(embed\right)$$

where embed denotes patch embedding, $\;embed^{"}$ denotes output after attention and normalization, and $\;embed^{"}"$ denotes output after the feedforward process, residual addition, and normalization process.

#### 3.1.5 Class token

The class token is a special token that is added to the patch embedding sequence. This token’s final output is sent to the transformer encoder, which classifies the image.

#### 3.1.6 MLP head

**Figure 1:**
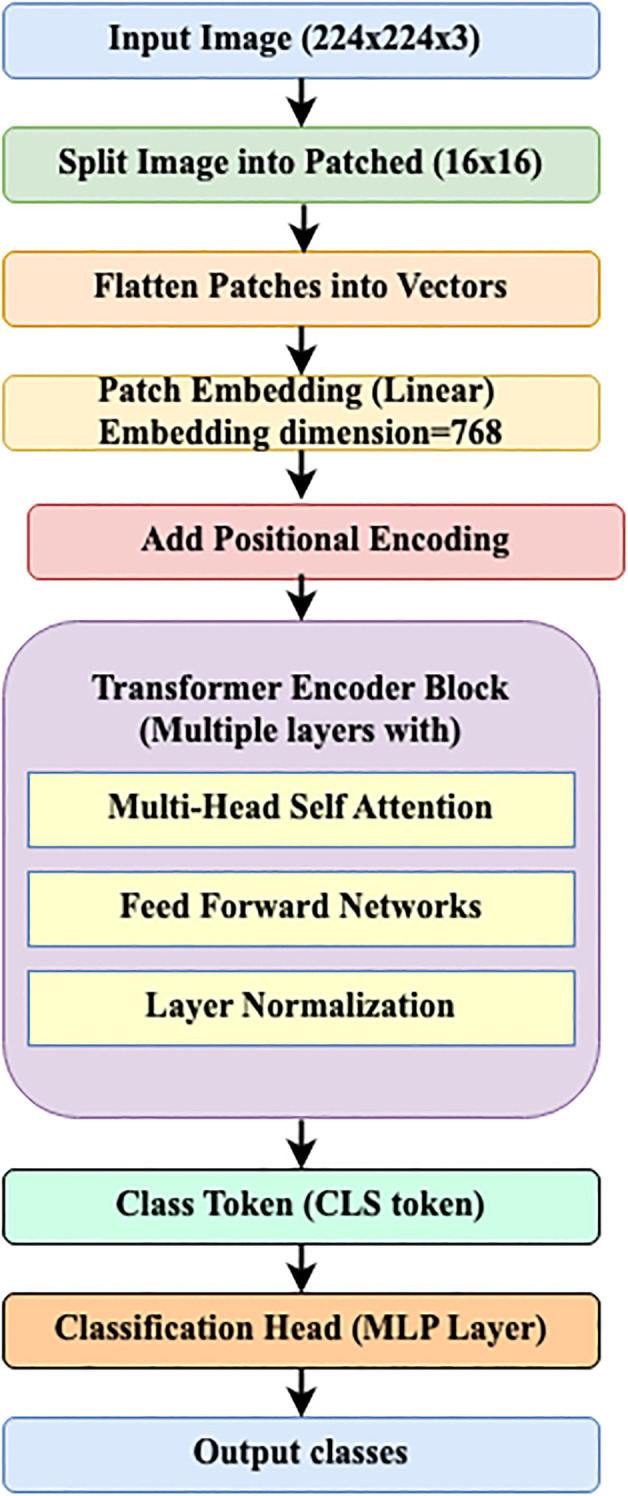
Overall process of vision transformer.

The class token’s output is given to the Multi-Layer Perceptron (MLP) head, which uses the soft max activation function to provide the final class prediction. **Figure 1** depicts the whole process of a vision transformer.

### 3.2 ResNet18

The pre-trained ResNet18 model receives the image as input. It starts with an initial convolutional layer that applies both convolution and max pooling.

#### 3.2.1 Input image

The convolution layer divides the image into blocks of 7x7 using a 64-size filter and a stride of 2, and its calculation is shown in Equation 13.

(13) $$out=Conv2D\left(in,w_{1},b_{1}\right)$$

where in and out represent the input and output of the convolution layer, respectively. $w_{1}\;and\;b_{1}$ denote weights and bias convolution filter.

#### 3.2.2 Max pooling

The max pooling layer processes a 3x3 input feature map and selects the maximum value.

The stride value for this operation is set to 2, as represented in Equation 14.

(14) $$out_{pooled}=MaxPool\left(out,\;Kernel\;size=3,\;stride=2\right)$$

where $out_{pooled}$ denotes output of the pooling layer.

#### 3.2.3 Residual block

ResNet18 has 4 layers, including residual blocks near the first convolution layer. Layer1 has two residual blocks with two convolutional layers. Each layer of the 3x3 input feature map includes 64 filters and stride 1. Two remaining bricks form Layer2. Two convolutional layers make up each residual block, and the input feature map is 3x3. Every layer has 128 filters and a stride of 2. Two leftover blocks make up Layer 3. Two convolutional layers and a 3x3 input feature map make up each residual block. Each layer has 256 filters and a stride of 2. Two blocks remain in layer 4. Each residual block comprises two convolutional layers, 512 filters, a 3x3 input feature map, and a stride of 2. The operations of the residual block are typically expressed in Equations 15–17.

(15) $$IFM=ReLu\left(Conv2D\left(in,\;w_{2},b_{2}\right)\right)$$

(16) $$IFM=ReLu\left(Conv2D\left(IFM,\;w_{3},b_{3}\right)\right)$$

(17) out=ReLu(in+IFM)

where IFM denotes intermediate feature map.

#### 3.2.4 Global average pooling

After the final residual blocks, a 7x7 Global Average Pooling (GAP) layer is added to reduce the feature dimensions from 3x3 to 1x1. The feature map of GAP is presented in Equation 18.

(18) $$out_{GAP}=\frac{1}{Height\times Weight}\sum_{i=1}^{Height}\sum_{j=1}^{Width}out\left[i,j\right]$$

where out denotes the output of each residual block.

#### 3.2.5 Fully connected layer

The global average pooling output is provided to the FC layer for class prediction, as indicated in Equation 19.

(19) $$Z=out_{GAP}\;\times w_{4}+b_{4}$$

where Z denotes the logit function i.e., before applying the Softmax function, $w_{4}$ and $b_{4}$ are the weight matrix and the bias matrix.

#### 3.2.6 Softmax layer

The final class probability is determined through the Softmax layer, with the calculation provided in Equation 20.

(20) $$Prob_{i}=\frac{e^{z_{i}}}{\sum_{j=1}^{n}e^{z_{j}}}$$

where $Prob_{i}$ denotes probability of i^th^ class and $z_{i}$ denotes logits for the ith class.

**Figure 2:**
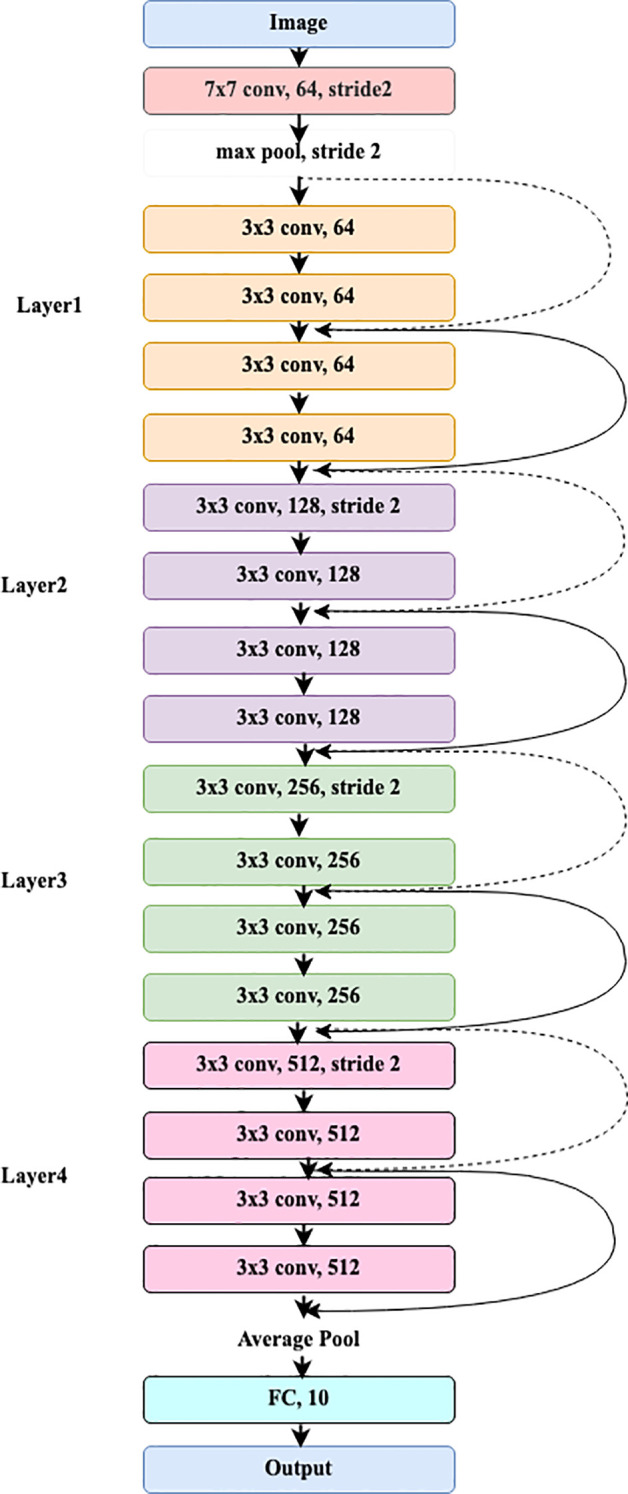
Overall process of ResNet18 model.

**Figure 2** depicts the complete workflow of the ResNet18 model.

## 4 The proposed hybrid vision transformer with ResNet18 model

This work proposes a hybrid Vision Transformer (ViT) with ResNet18 pre-trained models (ViT-ResNet18). The input images are processed independently using the pre-trained ViT and ResNet18 models. The output features of these two models are concatenated and provided to the final FC layer.

**Figure 3:**
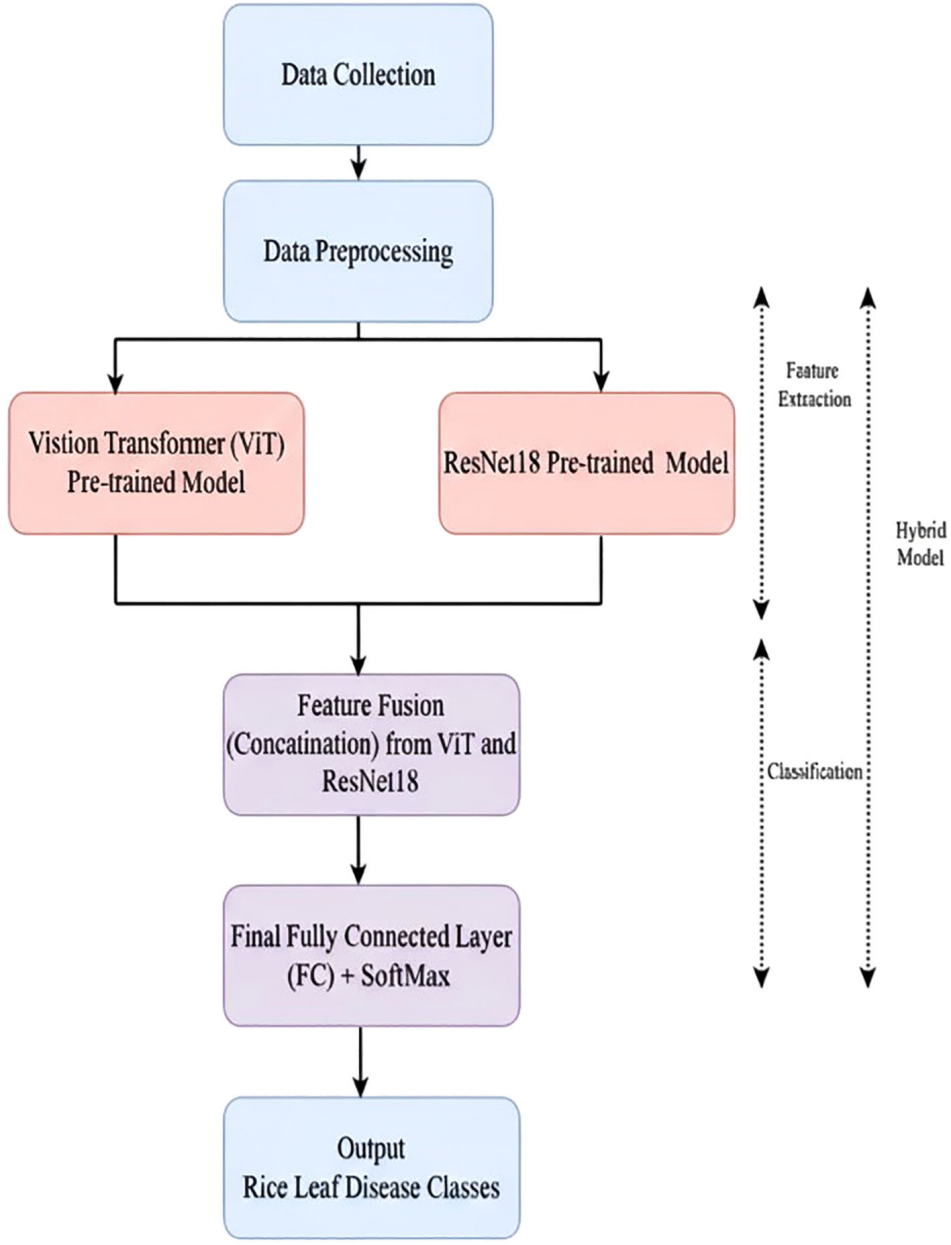
Proposed hybrid vision transformer (ViT)-RestNet18 model.

A Softmax layer is subsequently applied to the FC output to perform the classification of rice leaf diseases. The comprehensive workflow of the Hybrid ViT-RestNet18 Model is illustrated in **Figure 3**.

### 4.1 Data collection

Diseases in the rice plant impact various parts of the body. The dataset has been sourced from publicly accessible repositories, specifically Kaggle (Rice diseases image dataset, 2024) and Mendeley (Rice leaf diseases dataset, 2023). The dataset for rice leaf disease available on Kaggle comprises a total of 3,345 images, categorized into four distinct classifications. The Brown spot class contains 523 images, the Healthy class consists of 1,488 images, the Hispa class includes 565 images, and the Leaf Blast class has 779 images. The Mendeley rice leaf disease dataset consists of 4,684 images organized into three separate categories. The Bacterial blight class contains 1604 images, the Brown spot class comprises 1620 images, and the Leaf smut class consists of 1460 images. This work considers six classes derived from two distinct datasets. **Table 2** presents the specifics of the combined dataset, detailing each class along with its corresponding image distribution.

**Table 2:** Class-wise data distribution in the combined dataset.

Disease name	Image count
Healthy	1488
Hispa	565
Bacterial Blight	1604
Brown Spot	1620
Tungro	1308
Leaf Blast	779

### 4.2 Data preprocessing

**Figure 4:**
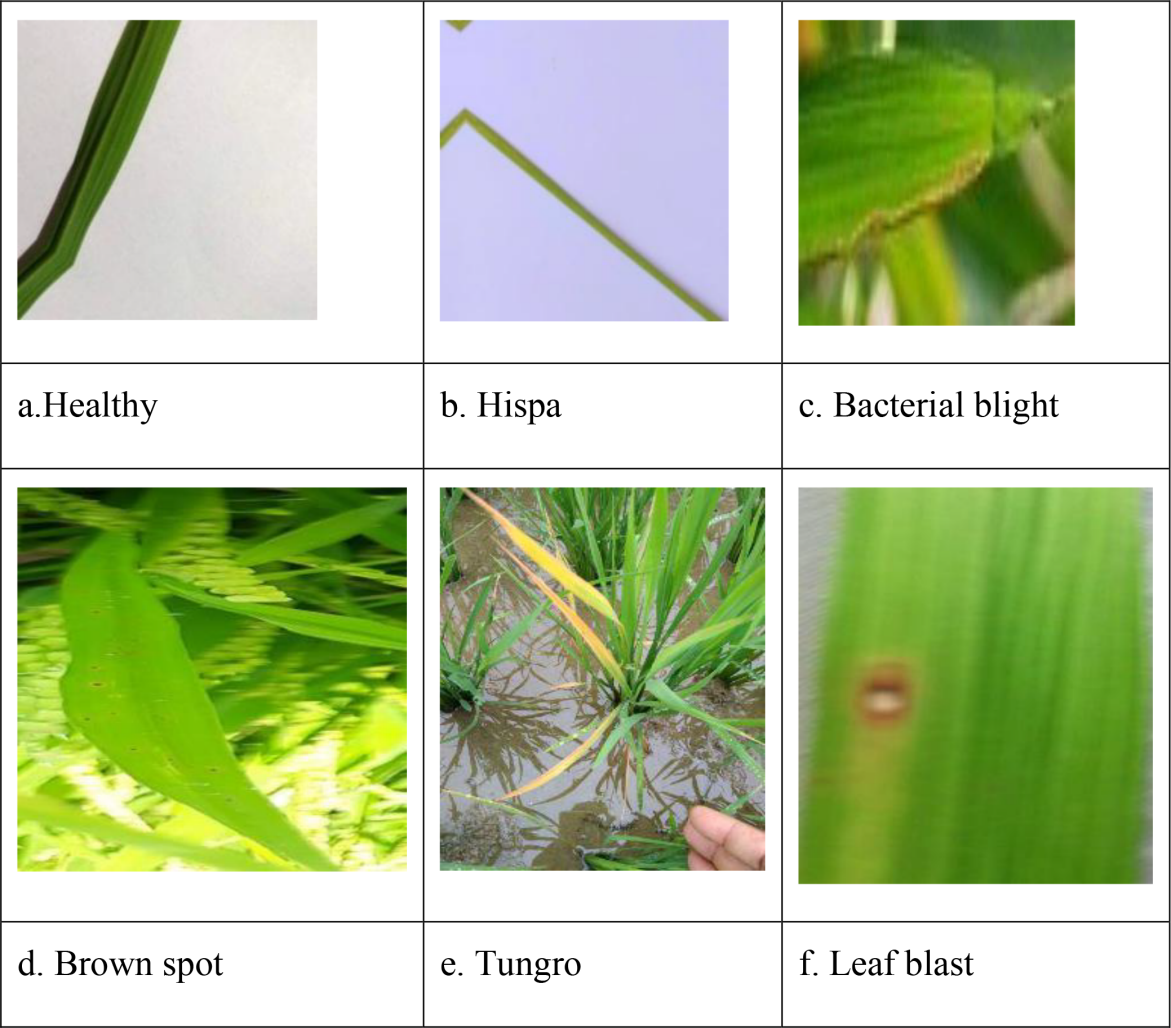
Sample images of rice leaf diseases: **(a)** Healthy, **(b)** Hispa, **(c)** Bacterial blight, **(d)** Brown spot, **(e)** Tungro, and **(f)** Leaf blast.

Image augmentation utilizes the Albumentations library to achieve a balanced distribution of images across all classes, as specified in the base paper by Kaur et al (Kaur et al., 2024). The augmentation process encompasses several operations, including horizontal and vertical flipping, blurring, random adjustments of brightness and contrast, as well as shifting, scaling, and rotating. **Figure 4**. Shows the sample image of rice leaf diseases namely, Healthy, Hispa, bacterial Blight, Brown Spot, Tungro and Leaf Blast. **Table 3** presents the distribution of data categorized by class for rice lease diseases.

**Table 3:** class-wise data distribution for rice lease diseases.

Disease name	Image count
Healthy	2382
Hispa	904
Bacterial Blight	1584
Brown Spot	1600
Tungro	1308
Leaf Blast	1440

### 4.3 Feature extraction and classification

#### 4.3.1 Input preparation

The input image is first resized to 224 × 224 pixels. It is then given as input to both the ViT and ResNet18 modules.

#### 4.3.2 Feature extraction with ViT

The model uses the ViT variant google/vit-base-patch16-224-in21k. The image is split into 16 × 16 patches. Each patch is flattened into a vector and passed through a linear embedding layer. Positional encoding is added to retain spatial information. The sequence of patch embeddings is then processed by transformer encoder layers. Each encoder applies multi-head self-attention and feed-forward layers to preserve global context. The final class token is extracted as the ViT feature vector.

#### 4.3.3 Feature extraction with ResNet18

The same input image is also processed by ResNet18, a convolutional neural network. The network uses convolutional layers, residual blocks, and pooling layers. These layers capture local features like edges, textures, and shapes. A global average pooling (GAP) layer then reduces dimensions and produces a feature vector.

#### 4.3.4 Feature fusion

The Vision Transformer (ViT) produces a feature vector that captures global image context. ResNet18 produces another feature vector that captures local patterns such as edges and textures. These two vectors are combined through concatenation to form a single feature representation.

Mathematically, if $F_{ViT}\in R^{d1}$ is the ViT feature vector and $F_{ResNet}\in R^{d2}$ is the Resnet18 feature vector, the fused feature is expressed in (Equation 21).

(21) $$F_{fusion}=\left[F_{ViT}\;\left|\;\right|F_{ResNet}\right]$$

where || denotes the concatenation operation.

This fused feature vector combines both global and local information, giving the model a richer representation of the rice leaf image. It is then passed to the fully connected layer for classification.

#### 4.3.5 Classification

The fused feature vector $F_{fusion}$ is sent to a fully connected (FC) layer. The FC layer maps this high-dimensional feature into class scores, also called logits. The FC operation can be written in Equation 22.

(22) $$Z=W.F_{fusion}+b$$

where W is the weight matrix, b is the bias term, and Z is the logit vector for all classes.

The logits are then passed through a Softmax function to convert them into probabilities. For a class ‘*i*’, the probability is calculated as shown in Equation 23.

(23) P(y=ix)=eZi∑j=1CeZj

where C is the total number of classes and $Z_{i}$ is the logic value for ‘i’.

The final output is the class with the highest probability, predicting the type of rice leaf disease.

#### 4.3.6 Output

The final classification predicts one of six classes: healthy, hispa, bacterial blight, brown spot, tungro and leaf blast.

## 5 Results and discussion

The proposed Hybrid ViT–ResNet18 model is compared with three existing models: VGG16 with Neural Network, Inception V3 with Neural Network and Squeeze Net with Neural Network (Kaur et al., 2024). The authors discuss the accuracy and loss of the proposed model utilizing training and validation data, the confusion matrix based on training data and test data, and will conclude with an evaluation of performance using specified metrics.

### 5.1 Model parameters and values

In this work, the dataset is randomly split into 70% of training data and 30% of testing data, similar to the method used by Kaur et al (Kaur et al., 2024). The dataset consists of six categories: healthy, hispa, bacterial blight, brown spot, tungro, and leaf blast. All input images are accurately classified and organized into their respective categories. The proposed hybrid ViT-ResNet18 model incorporates the following essential parameter values and assumptions. The total count of epochs is 17. The variant of the ViT model has been named as google/vit-base-patch16-224-in21k. The ResNet variant is ResNet18. It is used Adam optimization algorithm, configured with a learning rate of 0.001 and a batch size of 32. **Table 4** shows the parameters and hyperparameter values of the proposed hybrid ViT–ResNet18 Model.

**Table 4:** Parameters and hyperparameter values of the proposed hybrid ViT–ResNet18 model.

Parameter/hyperparameter	Value/setting
Dataset split	70% training, 30% testing
Number of classes	6 (Healthy, Hispa, Bacterial blight, Brown spot, Tungro, Leaf blast)
Image size	224 × 224 pixels
Vision Transformer variant	google/vit-base-patch16-224-in21k
ResNet variant	ResNet18
Number of epochs	17
Optimizer	Adam
Learning rate	0.001
Batch size	32
Loss function	Cross-entropy loss

### 5.2 Experimental setup

This study was performed on a system equipped with an Intel i7 CPU, NVIDIA RTX 3090 GPU, and 16 GB of RAM. The hybrid ViT-ResNet18 model was built using NumPy, Pandas, Python, and PyTorch 2.0.

### 5.3 Ablation study

In order to analysis the role of each component of the hybrid architecture suggested, an ablation study between two variations of model, (1) Hybrid ViT-ResNet18, and (2) Hybrid ViT-ResNet18 accompanied by Neural Network, was performed as a comparative study. The aim of comparing both variations of model here is to identify how adding one more Neural Network layer following the feature fusion process impacts the classification ability of a model.

**Table 5** shows quantitative outcome of both variants on several evaluation criteria, AUC, Accuracy, F1-Score, Precision, and Recall.

**Table 5:** Performance comparison of hybrid ViT-ResNet18 with neural network and hybrid ViT-ResNet18.

Evaluation metrics	Hybrid ViT-ResNet18 with neural network	Hybrid ViT-ResNet18
AUC	0.984	0.985
Accuracy	0.935	0.944
F1-Score	0.930	0.942
Precision	0.930	0.942
Recall	0.933	0.942

From **Table 5**, it is clear that the Hybrid ViT-ResNet18 model moderately dominates its version based on the Neural Network layer by all evaluation criteria. The hybrid model reached an accuracy improvement of 0.6%, and parallel rise in F1-Score, precision, and by recall also. The slight difference of AUC (0.985 *vs*. 0.984) also implies that both models are highly discriminative, but simpler hybrid model based on omission of Neural Network layer generalizes better.

These results recommend that incorporating an extra Neural Network layer does not greatly improve model performance and potentially adds bloat. The better performance of the Hybrid ViT-ResNet18 shows that it is possible to achieve direct ViT-ResNet18 feature representation fusion and a fully connected classifier for effective capture of rice leaf disease image discriminative patterns, leading to more compact and precise classification.

### 5.4 Model accuracy and loss

**Figure 5:**
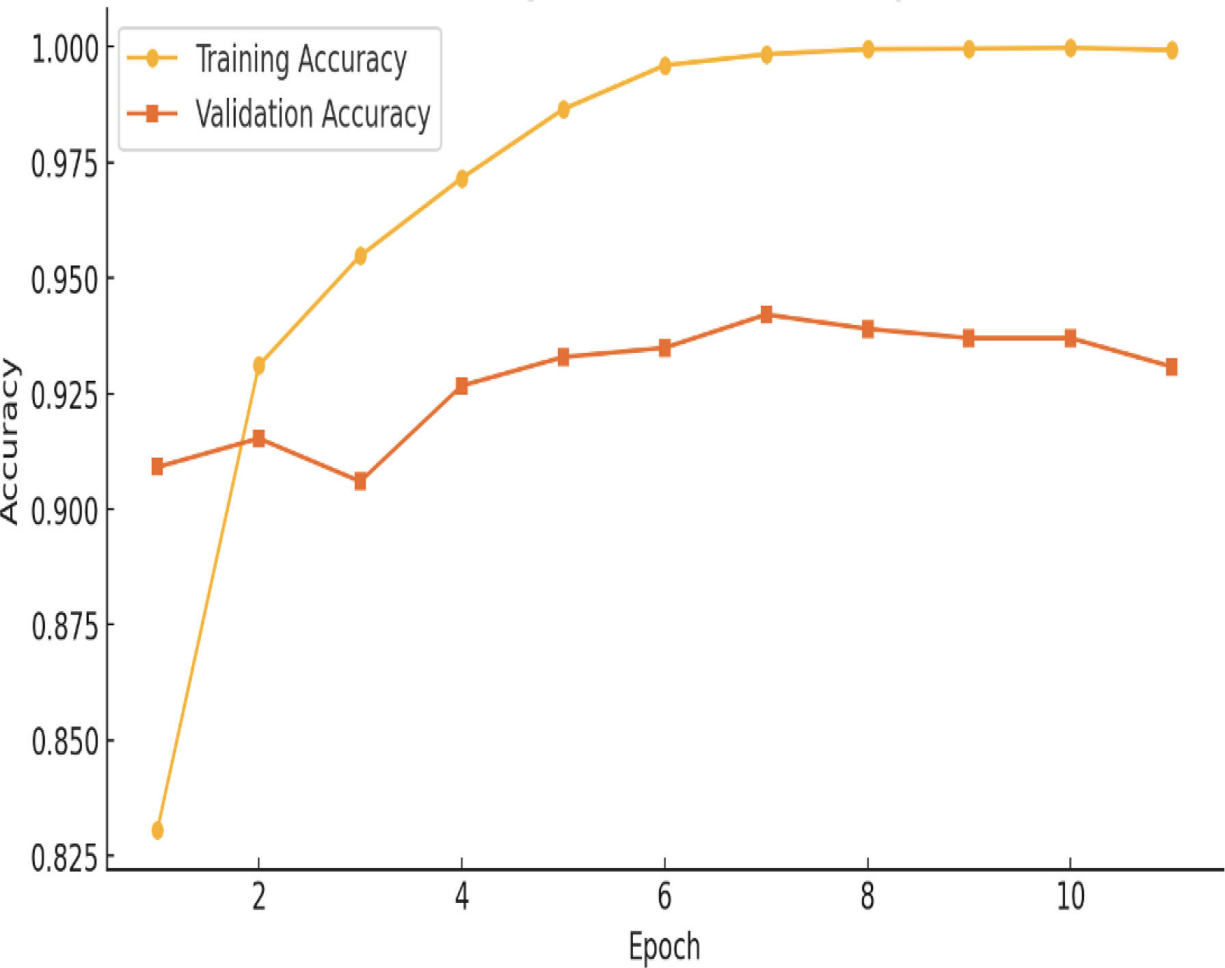
Hybrid ViT with ResNet18 training *vs* validation accuracy.

**Figure 5** illustrates the accuracy of the proposed hybrid Vit-ResNet18 model, comparing both training and validation datasets. Epochs are shown on the x-axis. Accuracy of the model, from 0 to 1, is shown on the Y-axis. The yellow color data points and line represent the training accuracy for the proposed model. The initial observation indicates a lower model accuracy, which subsequently shows a gradual increase over time. The accuracy has remained consistent, exhibiting the same values from epoch 8 through epoch 17. The orange color data points and lines represent the validation accuracy. During the initial three epochs, significant fluctuations in accuracy were observed. Subsequently, the accuracy values stabilized, consistently remaining above 90%.

**Figure 6:**
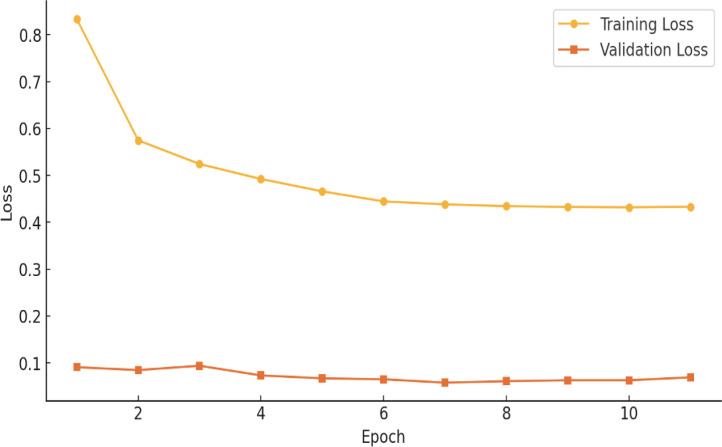
Hybrid ViT-ResNet18 training *vs* validation loss.

**Figure 6** illustrates the loss of the proposed hybrid ViT-ResNet18 model, plotted against both training and validation datasets. Epochs are shown on the x-axis. Loss of the model, from 0 to 1, is shown on the Y-axis. The yellow color data points and line represent the training loss associated with the proposed model. The initial observation indicates that the model loss remains elevated for the first two epochs. The model loss has subsequently shown a gradual decrease. The loss values have remained consistent from epoch 6 through epoch 17. The orange color data points and lines represent the validation loss. The validation loss is recorded at approximately 10%.

### 5.5 Performance evaluation metrics

Accuracy, precision, recall, F1-score, and the confusion matrix were some of the measures used to evaluate the models that were concentrated on DL.

#### 5.5.1 Accuracy

This ratio compares the number of correct predicts to the total number of predictions. The calculation is given in Equation 24.

(24) $$\text{Accuracy=}\frac{\text{Number of right prediction}}{\text{Total number of prediction}}$$

#### 5.5.2 Precision

It quantifies the number of positive predictions that correspond to actual positive instances. The calculation is presented in Equation 25.

(25) $$\text{Precision=}\frac{Number\;of\;True\;Positive}{Number\;of\;True\;Positive+\;Number\;of\;False\;Positive}$$

#### 5.5.3 Recall

The metric quantifies the number of actual positive instances that were accurately predicted by the proposed model. The calculation is provided in Equation 26.

(26) $$\text{Recall=}\frac{Number\;of\;True\;Positives}{Number\;of\;True\;Positives+Number\;of\;False\;Negatives}$$

#### 5.5.4 F1-score

Precision and recall are used to evaluate model performance. The calculation is presented in Equation 27.

(27) $$F1-Score=\frac{2\times P\times R}{P+R}$$

where P and R indicate precision and recall.

### 5.6 Model performance evaluation

**Figure 7:**
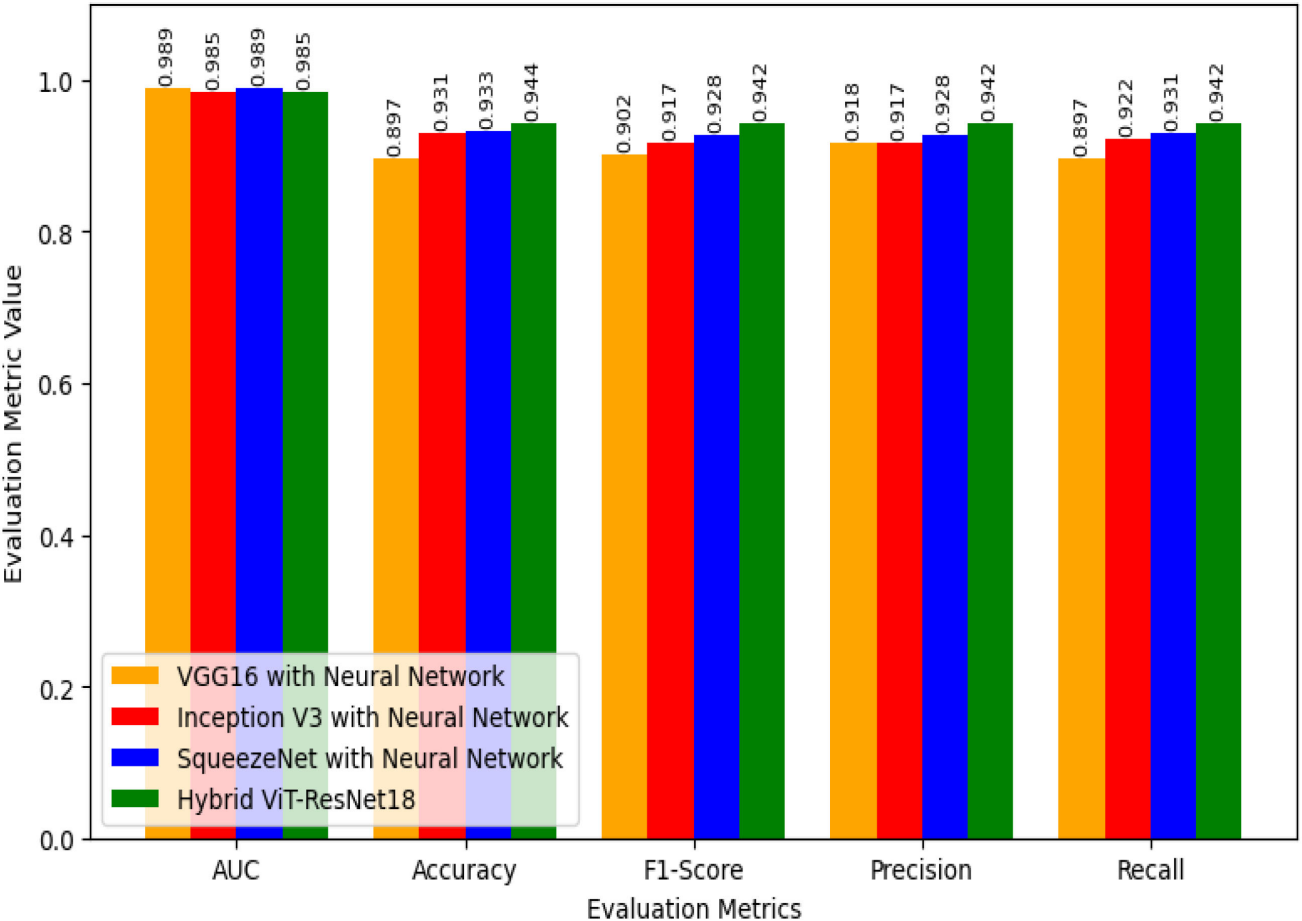
Performance evaluation of proposed hybrid ViT-ResNet18 and existing models.

**Figure 7** shows how four models perform. These are VGG16 with Neural Network, Inception V3 with Neural Network, SqueezeNet with Neural Network, and the proposed Hybrid ViT-ResNet18. Each model is tested under the same setup to compare accuracy and reliability. The Hybrid ViT with ResNet18 stands out by offering stronger results and better feature learning.

For the Area Under the Curve (AUC), both VGG16 with Neural Network and SqueezeNet with Neural Network score 0.989. Inception V3 with Neural Network and the Hybrid ViT with ResNet18 record slightly lower scores of 0.985. Although the Hybrid ViT-ResNet18 falls a bit short in AUC, it performs better in other key measures. This shows that the Hybrid model is strong overall and handles tasks more effectively than the others.

For accuracy, VGG16 scores 0.897, Inception V3 scores 0.931, and SqueezeNet reaches 0.933. The Hybrid ViT achieves the top score of 0.944, which is 1.1% higher than SqueezeNet and 4.7% higher than VGG16.

The F1-Score results follow a clear trend. VGG16 with Neural Network scores 0.902, Inception V3 with Neural Network reaches 0.917, and SqueezeNet with Neural Network achieves 0.928. The proposed Hybrid ViT-ResNet18 outperforms them all with an F1-Score of 0.942, showing a stronger balance between precision and recall.

Regarding Precision, VGG16 with Neural Network achieves 0.918, Inception V3 with Neural Network records 0.917, and SqueezeNet with Neural Network reaches 0.928, whereas the Hybrid ViT-ResNet18 attains 0.942, demonstrating an approximate 1.4% enhancement over the next best model.

For Recall, Neural Network with VGG16 marks 0.897, Neural Network with Inception V3 marks 0.922, Neural Network with SqueezeNet marks 0.931, whereas Hybrid ViT-ResNet18 marks highest recall of 0.942, hence marks better ability for proper identification of positive instances.

Overall, if we compare the AUC values of our suggested Hybrid ViT-ResNet18 to those of other models, it shows equivalent results, yet it shows steady improvement across accuracy, F1-score, precision, and precision, hence proving its reliability and better performance compared to classical CNN-based architectures. **Table 6** provides the efficacy evaluation of the proposed Hybrid ViT-ResNet18 and existing models.

**Table 6:** Efficacy evaluation of the proposed hybrid ViT-ResNet18 and existing models.

Evaluation metrics	VGG16 with neural network	Inception V3 with neural network	SqueezeNet with neural network	Hybrid ViT with ResNet18
AUC	0.989	0.985	0.989	0.985
Accuracy	0.897	0.931	0.933	0.944
F1-Score	0.902	0.917	0.928	0.942
Precision	0.918	0.917	0.928	0.942
Recall	0.897	0.922	0.931	0.942

**Figure 8:**
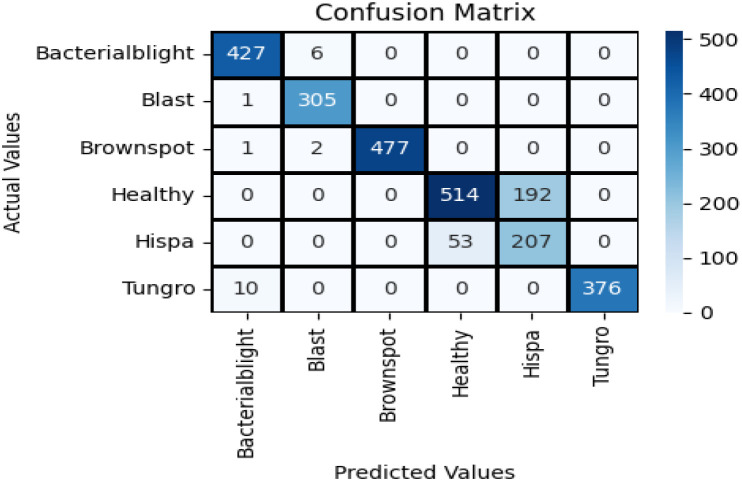
Confusion matrix of VGG16 with neural network.

The confusion matrix illustrates how well a classification model performs by showing the number of correct and incorrect predictions for each class. **Figure 8** shows the confusion matrix of VGG16 with Neural Network. It reached high accuracy for most classes. Bacterial blight had 427 correct out of 433, Blast had 305 out of 306, Brownspot had 477 out of 480, and Tungro had 376 out of 386. These results show the model can recognize clear disease patterns with few errors. The main problem was between Healthy and Hispa leaves. The model confused 192 Healthy samples as Hispa and 53 Hispa samples as Healthy. This happened because the two look very similar. While this reduced accuracy for those two classes, the model still outperformed many earlier methods. With more training data, stronger image features, or attention-based methods, the Healthy–Hispa confusion can be reduced. This would make the model more reliable for real use in farms and crop health monitoring.

The Inception V3 with Neural Network model gave strong results in classifying rice leaf diseases. It correctly identified most cases of Bacterial Blight (428 of 433), Blast (305 of 306), Brown Spot (477 of 480), and Tungro (376 of 386). The main weakness was in separating Healthy and Hispa leaves. In this case, 192 Healthy samples were marked as Hispa and 53 Hispa samples were marked as Healthy. This shows that the two classes share close visual traits, making them hard to tell apart. The model still learned clear disease patterns and gave steady results. With more training images and better handling of Healthy and Hispa, it could perform better in farm use. 

**Figure 9:**
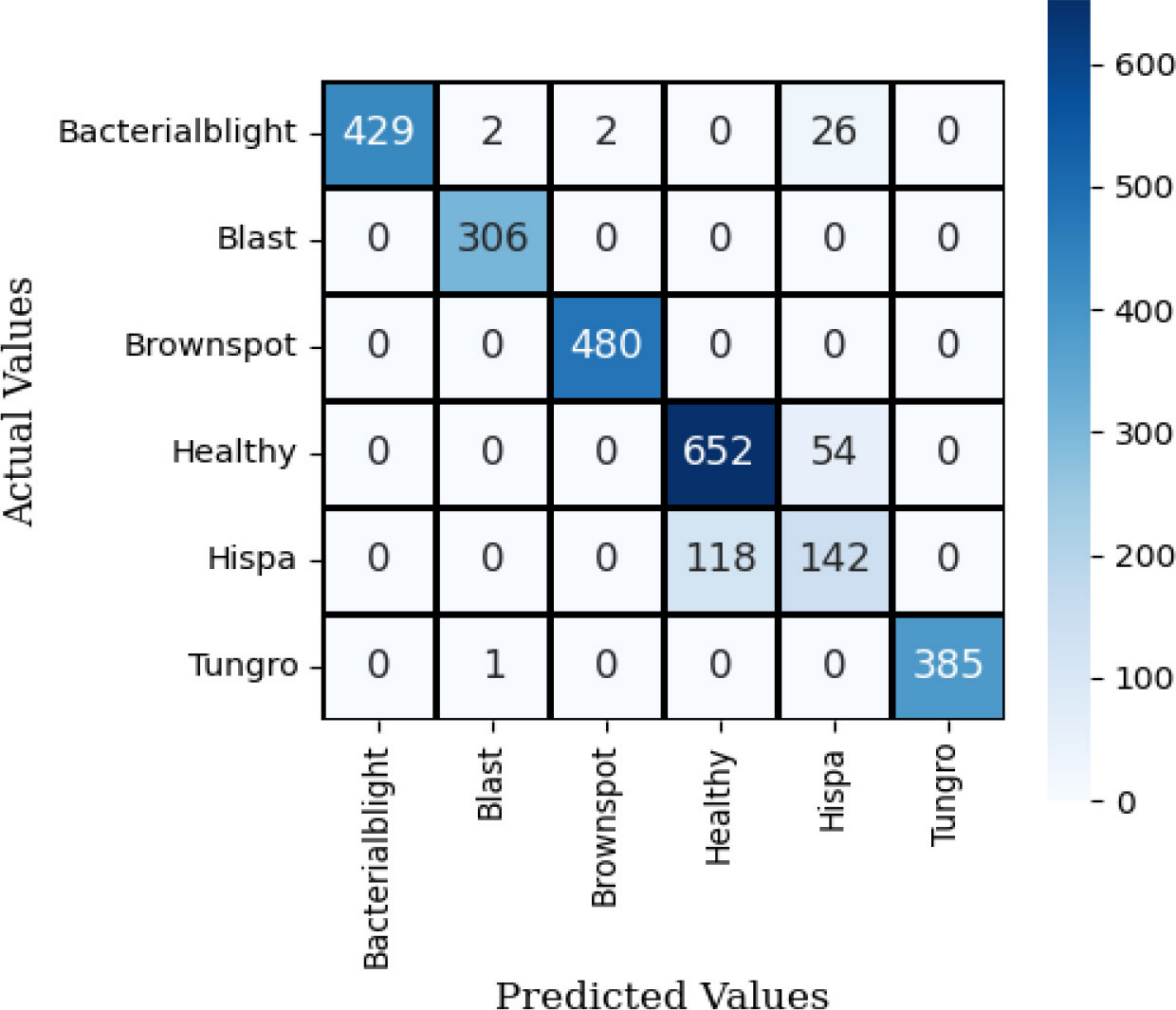
Confusion matrix of squeeze net with neural network.

The confusion matrix for SqueezeNet applied to the Neural Network using test data is shown in **Figure 9**. The observation indicates that the dark colors in the grid represent correct predictions, while the grids with a white background signify misclassifications of this model.

**Figure 10:**
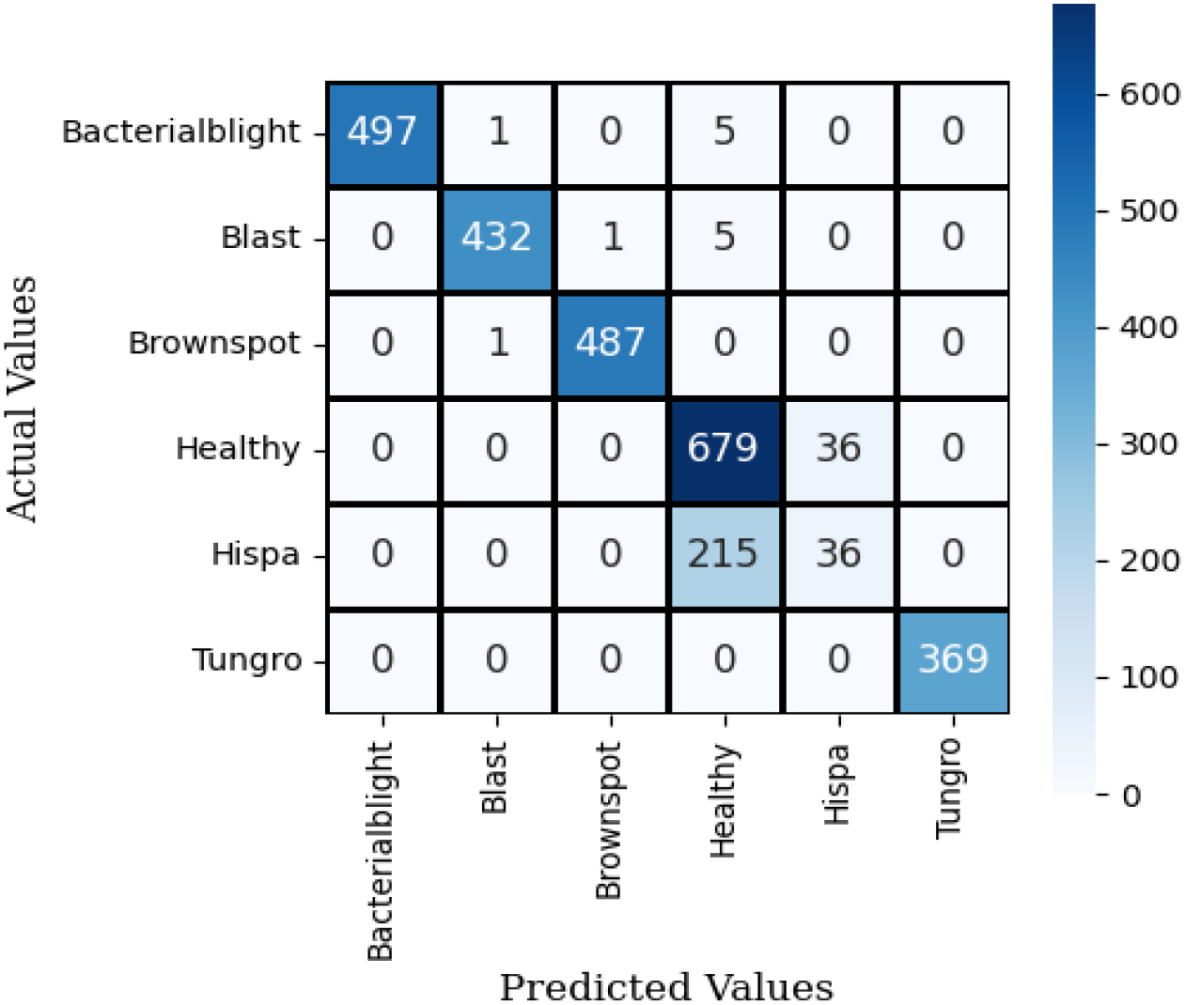
Confusion matrix of hybrid ViT-ResNet18.

**Figure 10** illustrates the confusion matrix for the proposed hybrid ViT with ResNet18, utilizing the test dataset. The observation indicates that the dark colors in the grid represent correct predictions, while the grids with a white background signify misclassifications of the model.

It is observed that the proposed Hybrid ViT integrated with ResNet18 demonstrates a higher accuracy in predictions compared to VGG16 with Neural Network, Inception V3 with Neural Network and SqueezeNet combined with a neural network.

## 6 Analysis and discussion

**Figure 11:**
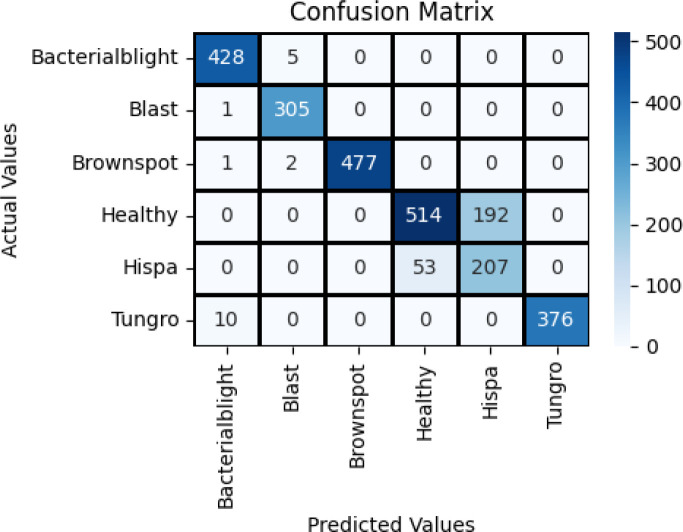
Confusion matrix of inception V3 with neural network.

The hybrid ViT-ResNet18 model was evaluated on the aspects of the accuracy, precision, recall, F1-score, and AUC as illustrated in **Figure 11**. The results obtained 94.4% accuracy, 0.948 precision, 0.944 recall, 0.942 F1-score, and 0.985 AUC, respectively. As presented, the results indicate that the model keeps a good equilibrium between sensitivity and specificity.

The performance of the hybrid model was better than that of baseline models. The accuracy was better than VGG16 with Neural Network by 5% and InceptionV3, SqueezeNet by 1%. This enhancement results from the combination of local and global features. The Vision Transformer are aware of overall image patterns, while fine details, along with texture information, are maintained by ResNet18. Combining them, feature learning, as well as misclassification reduction, are both enhanced.

The confusion matrix supports this conclusion. There are decent performances for all the classes of diseases, with less error for close classes. There are still some errors, however, particularly for classes with less number of images. This lends support for more extensive and balanced datasets. The hybrid ViT-ResNet18 model exhibits high confidence and robustness than the previously suggested techniques. Additional steps are expanding the dataset, validation with field images, and minimizing processing expense for real-time vision implementation.

## 7 Conclusion and future work

Agriculture is essential for sustaining human life. Detecting rice leaf disease is crucial for enhancing crop productivity. This work proposed a hybrid model that combines the Vision Transformer (ViT) design with a pre-trained ResNet18 to make predicting rice leaf diseases more accurate. The input images are processed independently using the pre-trained ViT-ResNet18 models. The output features of these two models are concatenated and provided to the final Fully Connected (FC) layer, followed by a Softmax layer for final classification. The output of rice leaf disease generated from the FC layer. The hybrid ViT-ResNet18 model has 94.4% accuracy, 0.948 precision, 0.944 recall, 0.942 F1-Score, and 0.985 AUC. The proposed hybrid model shows a 5%, 1%, and 1% improvement in accuracy compared to VGG16 with Neural Network, Inception V3 with Neural Network, and SqueezeNet with Neural Network classifier, respectively.

In future, more disease leaf classes will be incorporated to effectively address various rice leaf diseases. The results will be implemented in the mobile application. The application enables farmers to install it and automatically detect leaf diseases independently.

## Data Availability

The original contributions presented in the study are included in the article/supplementary material. Further inquiries can be directed to the corresponding author.
